# Supplementary material for: Marginal zone and follicular B cells respond differently to TLR4 and TLR9 stimulation
Source: bioRxiv. 2025 May 25:2025.05.20.655194. Preprint. [Version 1] doi: 10.1101/2025.05.20.655194 (PMC12139899; doi:10.1101/2025.05.20.655194)
Supplement: 1 [file NIHPP2025.05.20.655194V1-supplement-1.pdf]

bioRxiv preprint doi: https://doi.org/10.1101/2025.05.20.655194; this version posted May 25, 2025. The copyright holder for this preprint (which was not certified by peer review) is the author/funder, who has granted bioRxiv a license to display the preprint in perpetuity. It is made available under aCC-BY 4.0 International license.

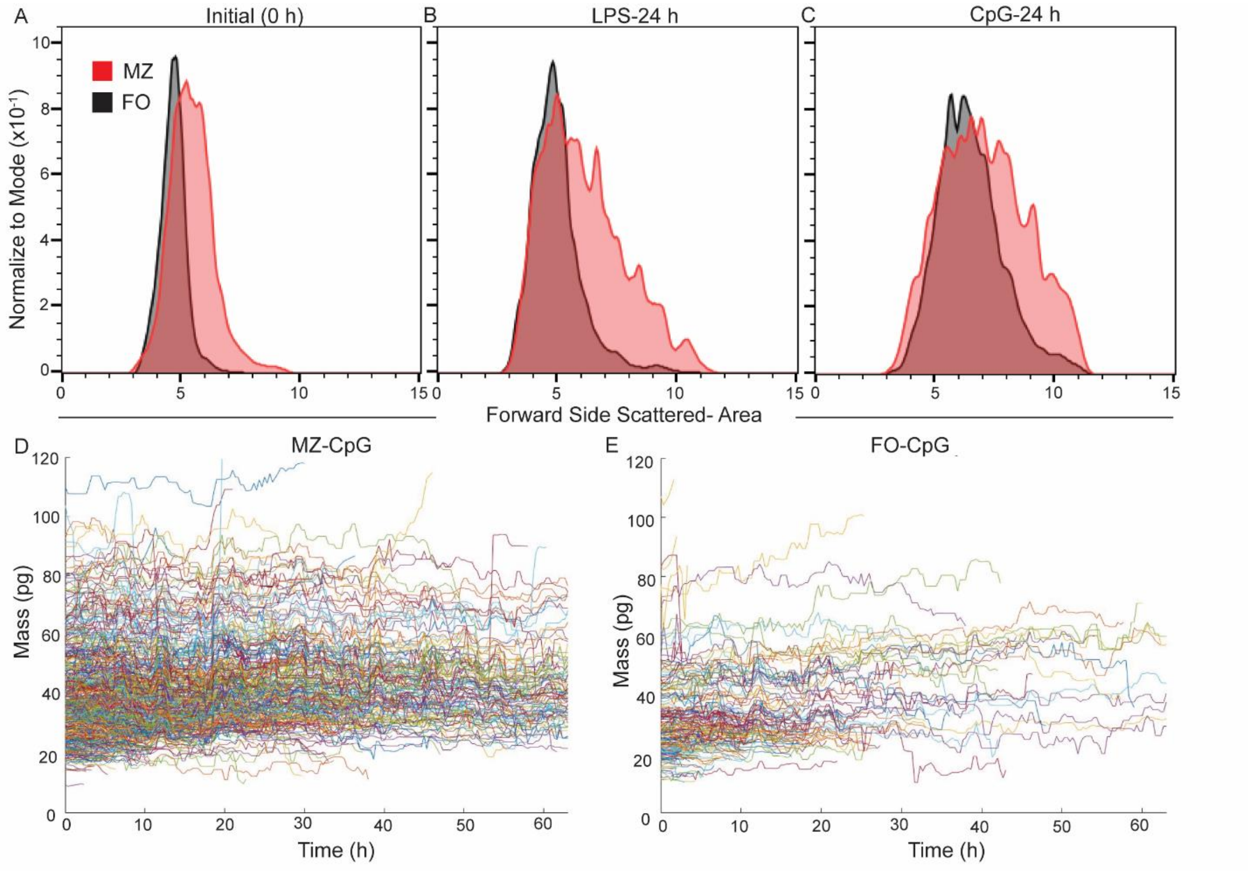


**Figure S1: MZ B cells are larger than FO B cells.** The size of MZ and FO B cells was measured using flow cytometry, as determined by forward-scattered light. **A)** size at 0 h, **B)** size at 24 h following LPS stimulation, and **C)** size at 24 h following CpG stimulation. The MZ and FO B cells are represented by red and black histograms, respectively. **D)** and **E)** Linear cell growth trajectories of CpG-stimulated MZ and FO B cells. The X-axis represents time in hours, and the Y-axis represents cell mass in picograms (pg).
